# Nested miRNA Secondary Structure Is a Unique Determinant of miR159 Efficacy in *Arabidopsis*

**DOI:** 10.3389/fpls.2022.905264

**Published:** 2022-06-02

**Authors:** Muhammad Imran, Tengfei Liu, Zheng Wang, Min Wang, Shulin Liu, Xinyan Gao, Anning Wang, Songfeng Liu, Zhixi Tian, Min Zhang

**Affiliations:** ^1^State Key Laboratory of Plant Cell and Chromosome Engineering, Institute of Genetics and Developmental Biology, Innovative Academy for Seed Design, Chinese Academy of Sciences, Beijing, China; ^2^Beijing Vegetable Research Center (BVRC), Beijing Academy of Agriculture and Forestry Sciences (BAAFS), Beijing, China; ^3^University of Chinese Academy of Sciences, Beijing, China

**Keywords:** *Arabidopsis thaliana*, pri-miR159a, nested miRNA structure, efficacy, duplex mutation

## Abstract

MicroRNAs (miRNAs) are 20- to 24-nucleotide small RNAs, and whenever a pri-miRNA precursor includes another miRNA precursor, and both of these precursors may generate independent non overlapping mature miRNAs, we called them nested miRNAs. However, the functional and regulatory roles of nested miRNA structures in plants are still unknown. In this study, the *Arabidopsis* nested miR159a structure, which consists of two nested miRNAs, miR159a.1, and miR159a.2, was used as a model to determine miRNA-mediated gene silencing in plants. Complementation analysis of nested miR159a structures revealed that the miR159a structure can differentially complement the *mir159ab* phenotype, and a duplex nested structure in the tail end region of the pre-miR159a fold back may have a possible dominant function, indicating the importance of the flanking sequence of the stem in the cleavage of the mature miRNA. Furthermore, continuously higher expression of the miR159a.2 duplex in the severe leaf curl phenotype indicates that miR159a.2 is functional in *Arabidopsis* and suggests that in plants, a miRNA precursor may encode multiple regulatory small RNAs. Taken together, our study demonstrates that the nested miR159a structure regulated by duplex mutations of miR159a has a unique pattern and provides novel insight into silencing efficacy of *Arabidopsis* miR159a.

## Introduction

MicroRNAs (miRNAs) are 20- to 24-nucleotide (nt) small RNAs that guide target mRNAs at the posttranscriptional level in both animals and plants (Jones-Rhoades et al., 2006). MiRNAs are also referred to as master regulators of gene expression in plants because they are active in several important developmental activities (Palatnik et al., 2003; Chen, 2012) and stress responses (Chiou et al., 2006; Sunkar et al., 2007). There are two separate stages in the plant miRNA pathway. First is the production stage, in which the microRNA is processed from a double-stranded RNA precursor transcript (Pegler et al., 2019). Second is the action stage, in which the mature microRNA guides an effector complex to directly suppress the expression of target genes that are highly complementary to microRNA target sequences (Schwab et al., 2005; Liu et al., 2014). It is obvious that both stages play an important role in the function of microRNAs. A number of studies have been performed on the action stage of the miRNA pathway, and many miRNA target mRNAs have been identified (Mallory et al., 2004; Schwab et al., 2005; Addo-Quaye et al., 2008). The microRNA production stage is complex and refers to many factors that take part in the progression to liberated mature microRNA. In addition to these factors related to microRNA production, a recent study proved that the secondary structure of pri-miRNAs has significant implications for miRNA processing (Starega-Roslan et al., 2011). For instance, pri-miR172a required an ~15-nucleotide fragment in the lower stem for processing (Mateos et al., 2010; Werner et al., 2010), while the upper stem area had only a minor effect. The efficiency and precision of miR171a and miR390a processing were aided by a bulge adjacent to a cleavage site in the lower stem of pri-miR171a and an area 4–6 nucleotides below the miR390a/miR390a* duplex (Cuperus et al., 2010; Song et al., 2010). In contrast, the conserved upper stem of pri-miR319, which includes a terminal loop, is critical for sequential cleavage for miR319 production, while the lower stem portion of pri-miR319 is unnecessary (Bologna et al., 2009). Similarly, other factor influencing the regulatory efficiency include miRNA precursors that produces fold back hairpin structures. A miRNA precursor is generally expected to produce one miRNA-miRNA* duplex (Bartel, 2004; Kim, 2005; Winter et al., 2009). Indeed, Zhang et al. reported that 19 miRNA precursors in Arabidopsis that can yield multiple distinct miRNA-like RNAs in addition to miRNAs and miRNA*s (Zhang et al., 2010). Therefore, functionally testing of these possible factors on miRNA-target interactions would be interesting.

*Arabidopsis* miR159 has been extensively examined as a framework for miRNA-mediated gene silencing in plants (Vella et al., 2004; Allen et al., 2010). *MIR159* is tightly involved in plant development and highly conserved in many plant species, including angiosperms, mosses, and lycopods (Rhoades et al., 2002; Li et al., 2014). Bioinformatics analysis predicted more than 20 targets potentially regulated by the deeply conserved miR159 family in *Arabidopsis*, including eight MYB genes. However, a genetic study showed that only *MYB33* and *MYB65* were functionally targeted by miR159, and *mir159ab* developmental abnormalities were reversed in a *myb33mir159ab* quadruple mutant (Allen et al., 2007). This limited functional precision, as established by genetics, has been discovered in other plant miRNA systems (such as the miR319 pathway), implying that additional factors are required for functional miRNA-target interactions (Seitz, 2009). However, one untested hypothesis is that whether nested miR159a.1 and miR159a.2 are sensitive to miR159 regulation in *Arabidopsis*. Recently, two reports efficiently advanced silencing efficacy. First, Zheng et al. reported that *MYB33/65* contains a distinctive RNA secondary structure (i.e., stem-loops overlapping the miR159 binding site) that is a sensitive target of miR159 (Zheng et al., 2017), which underpins the narrow functional specificity of Arabidopsis miR159. Second, Li et al. demonstrated that factors beyond complementarity (e.g., miR159:*MYB33* transcript stoichiometry and mutation of the miR159 binding site in *MYB33*) govern the efficacy of the miR159-*MYB33* silencing outcome (Li et al., 2014). This research contributes to the increasing evidence that sequence complementarity alone may not guarantee good miRNA silencing in plants and that other aspects may be functional.

In our deep-sequencing-based study of small RNAs we identified that many miRNA precursors (including *MIR159* precursors) were completely included in another miRNA precursor, and we called them nested miRNA structures (Liu et al., 2016). Previous research showed that the *MIR159* precursor is exceptionally long, and other tiny RNAs generated from it have been identified in *Arabidopsis* by large-scale sequencing (Fahlgren et al., 2007) and genome-scale analyses (Zhang et al., 2010; Li et al., 2011). In *Arabidopsis*, the miR159 family is encoded by three genes, *MIR159a*, *MIR159b*, *MIR159c*, and miR159a, and miR159b is highly expressed compared miR159c. miR159a and miR159b have redundant functions, and neither single mutant displayed any phenotype. The double mutant *mir159ab* has pleiotropic morphological defects, including altered growth habits, curled leaves, small siliques, and small seeds (Allen et al., 2007; Allen et al., 2010; Alonso-Peral et al., 2012; Allen et al., 2013; Li et al., 2014). Furthermore, two members, *ath-MIR159a* and *ath-MIR159b*, consist of sibling mature miRNAs, and another member, *ath-MIR159c*, does not consist of other mature miRNAs inside canonical miR159. Therefore, we used *ath-MIR159a* as a model to investigate the regulatory relationship of nested structures and miR159 functions. In this study, by carrying out in planta miR159 efficacy assays, to uncover the regulatory role of nested structure we hypothesized that (1) distant miR159a.1 regulate miR159a.2; (2) nested miR159a.2 regulate miR159a.1; (3) miR159a.1 or miR159a.2 itself regulated. To test these hypothesis, we restructured the *MIR159* precursors and analysed the effect of nested structure on miR159a function.

## Materials and Methods

### Plant Materials and Growth Conditions

*Arabidopsis thaliana* wild-type Columbia-0 (Col-0) and *mir159ab* mutant plants were grown in the greenhouse of the Institute of Genetics and Developmental Biology, Chinese Academy of Sciences. Seeds of the *mir159ab* mutant were provided by Professor Anthony A. Millar (Research School of Biology, Australian National University, Australia). Plants were grown in growth rooms with 16 h light and 8 h dark at 22°C. Size parameters were measured with ImageJ software.^1^

### Generation of Constructs Carrying Nested miR159a Structure Variants

For complementation of *mir159ab*, the various nested miR159a structure constructs *mir159a.1-solo*, *mir159a.1-rep*, *mir159a.2-solo*, *mir159a.2-rep*, *mir159a.12-invert*, *mir159a.123-mix*, *mir159a.12-mix*, *mir159a.13-mix*, *mir159a.23-mix*, *mir159a.21-mix*, and *mir159a.ns* containing a 186-bp genomic fragment of miR159a with respective duplex mutations were synthesized and cloned into the pUC57 vector by Integrated DNA Technologies, Beijing China. Synthesized fragments were sequenced to verify their integrity and then cloned into the pMDC99 vector (Invitrogen). To combine the promoter of miR159a, the 1.5 kb promoter region was amplified and cloned into the pMDC99 vector. Restriction enzyme digestion and sequencing were used to validate the entry vectors, which were then recombined into the destination vector pMDC99 through Gateway LR reaction (Curtis and Grossniklaus, 2003). Primers specific for each construct were designed by Primer Premier 5.0 (PREMIER Biosoft Palo Alto CA United States) and are listed in Supplementary Table S1.

### Transformation of *Arabidopsis*

All vectors were transformed into *Agrobacterium tumefaciens* strain GV3101 by electroporation (Hellens et al., 2000) and then transformed into the *Arabidopsis mir159ab* double mutant by using the floral dip method (Steven and Andrew, 1998). *A. tumefaciens* cells containing nested miRNA variants were harvested by centrifugation at 5000 × *g* for 8 min and resuspended in 5% sucrose solution to a final OD of 0.5. The shoot apex of *mir159ab* plants were dipped into a bacterial suspension supplemented with 0.05% Silwet (Silwet L-77, Sigma). Seeds were grown on agar plates containing Murashige and Skoog basal medium and antibiotics to pick transformants. Transformants were identified and transplanted into the soil after 7–10 days of growth.

### Quantitative Real-Time Polymerase Chain Reaction (qRTPCR) Assay

Total RNA was isolated from plants at different growth stages with TRIzol reagent (Invitrogen, United States), and the integrity of purified RNA was then examined by agarose gel electrophoresis and quantified with a Nanodrop 2000 spectrophotometer (Thermo Fisher Scientific, Waltham, MA, United States). For quantitative detection of the genes, cDNA was first synthesized using M-MLV (Promega, United States) and detected with TransStart Tip Green qPCR SuperMix (Trans Gen Biotech, China). For comparison of sibling mature miRNA, total RNA was purified with the miRcute miRNA purification kit (Tiangen Biotech, China) and reverse transcribed to cDNA with a miRcute Plus miRNA first-strand cDNA synthesis kit (Tiangen). A minute Plus miRNA qPCR kit (SYBR Green) was used for qRT-PCR by following the manufacturer’s protocol in a total volume of 20 μl. All qRT-PCRs (for both reference and genes of interest) were carried out on a Rotor-Gene Q Real-time PCR machine in triplicate under the following cycling conditions: 1 cycle of 95°C/5 min, 45 cycles of 95°C/15 s, and 60°C/15 s, and fluorescence was analysed at 72°C/20 s. A 55°C–99°C melting cycle was then carried out. *CYCLOPHILIN* (*At2g29960*) was used to normalize mRNA levels, and all sibling mature miR159 levels were normalized to U-6. The value for each gene represents the average of triplicate assays. The 2^−ΔCt^ method for relative quantification of gene expression was used to determine the level of miRNA expression.

### Statistical Analysis

The one-way ANOVA with the SPSS 11.5 package for Windows (SPSS, Inc., Chicago, IL, United States) was used for statistical analysis in this work. The Student’s *t*-test was used to examine the differences between the different groups of data. Results with a corresponding probability value of *p* < 0.05 and *p* < 0.01 were considered to be statistically significant and very significant, respectively.

## Results

### Effects of Nested pri-miR159a Structure Regulation

**Figure 1:**
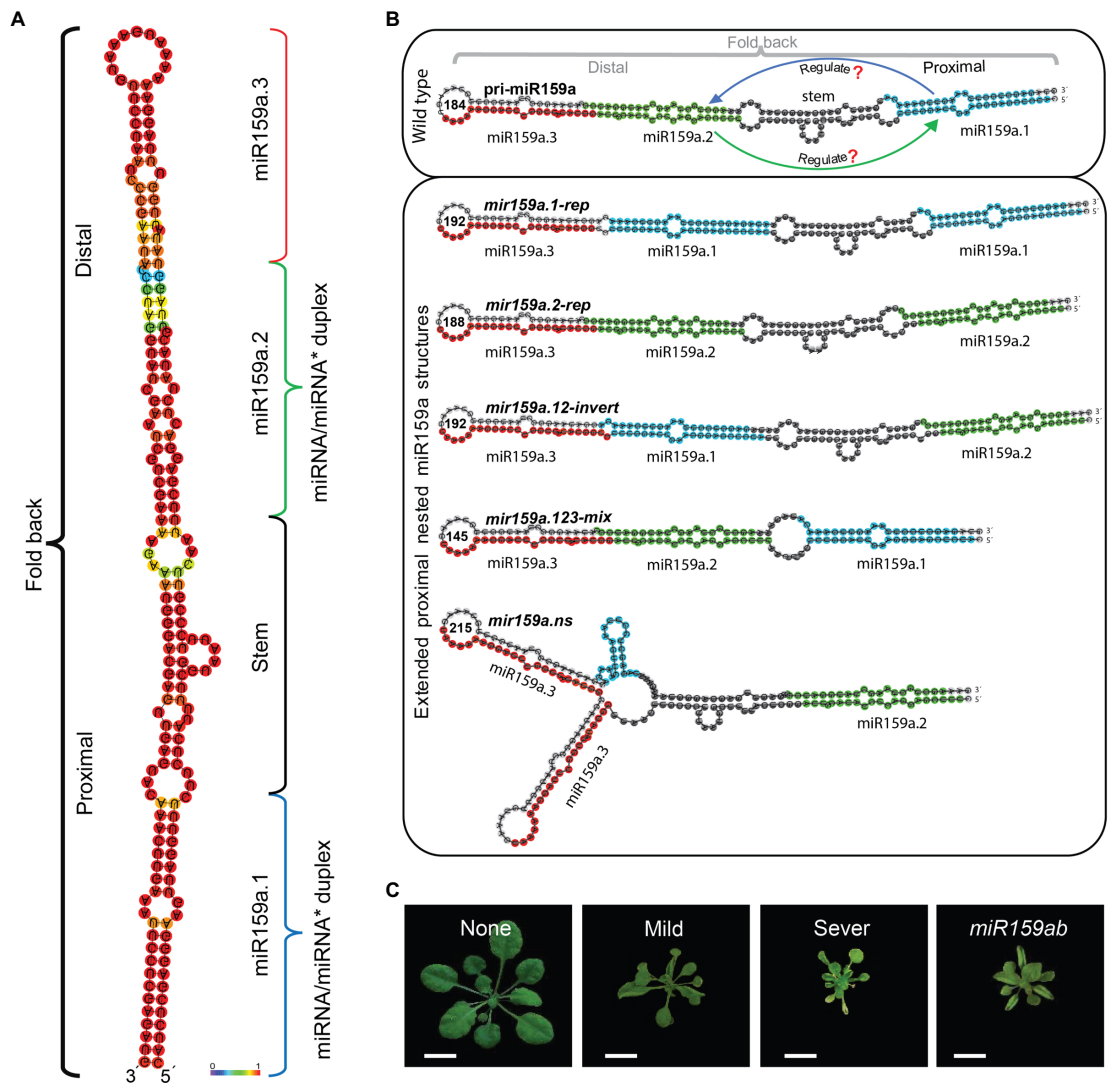
Effects of nested pri-miR159a structure and phenotype classification. **(A)** The secondary structure of the miR159a was predicted with mfold 3.2. Fold back indicates the proximal and distal parts used for pri-miR159a duplex mutation. Blue, green, and red colour indicate the position of duplex miR159a.1 (containing sibling’s miR159a.1–5 and miR159a. 1–3, that are located on 5 and 3 prime arm of miR159a secondary structure, respectively), miR159a.2 (Containing sibling’s miR159a.2–5 and miR159a. 2–3, that are also located on 5 and 3 prime arm of miR159a secondary structure, respectively) and miR159a.3, respectively. **(B)** Modified secondary structural view for pri-miR159a (upper box) set with comparison to different artificially designed variants (lower box) for complementation test in the extended proximal region. Blue, green, red, and black region on miRNA secondary structure indicates, miR159a.1, miR159a.2, miR159a.3, and core stem region, respectively. The green and blue arrow indicates that miR159a.1 may regulate miR159a.2 and vice versa (upper box). Each nested variant is named according to the combination of miRNA duplex region. **(C)** Three-week-old primary transformants from each construct grown in parallel and categorized based on the presence and severity of upward leaf curl (none; indistinguishable from wild-type, mild; display of less leaf curliness, and severe; more leaves showing curliness).

The processing of miRNA precursors results in the release of a double-stranded miRNA/miRNA* duplex. When a pri-miRNA precursor includes another miRNA precursor and both of these precursors may generate independent non overlapping mature miRNAs, we designated them nested miRNAs. In our deep-sequencing-based study of small RNAs, we identified that many miRNA precursors were completely included in another miRNA precursor (Liu et al., 2016). Among these miRNA precursors, *Phaseolus vulgaris* miR159a precursor encodes a second miR159a.2 (*Glycine max* precursor) and both have distinct accumulation pattern, independent of miR159 activity and does not preserve a direct relationship under different growth conditions, which suggests that pvumiR159a precursor can potentially introduce two distinct miRNAs expressed from same precursor (Contreras-Cubas et al., 2012) Furthermore, the *Arabidopsis* miR159a precursor is unusually long (Figure 1A) and additional small RNAs originating from the precursors have been reported through large scale sequencing (Fahlgren et al., 2007) and genome-scale analyses (Zhang et al., 2010; Li et al., 2011), but its regulatory role is still hidden. Therefore, to understand the important role of *Arabidopsis* nested miR159a structure, (which consists of three nested miRNAs, miR159a.1, miR159a.2, and miR159a.3) we hypothesized that (1) distant miR159a.1 can regulate miR159a.2; (2) nested miR159a.2 can regulate miR159a.1; (3) miR159a.1 or miR159a.2 itself can regulate (Figure 1B). To test this hypothesis, we artificially designed five nested miR159a structural constructs through miRNA duplex mutation, and analysed the effects of each precursor miRNA (miR159a.1, miR159a.2, and miR159a.3). To investigate the regulatory role of miR159a.1 and miR159a.2, first we designed the repeat of miR159a.1 (miR159a.1-rep) and miR159a.2 (miR159a.2-rep) constructs independently to identify the distant effect and strength. Then, to check the structural position influence on regulation of miR159a.1 and miR159a.2, we exchanged the position between these two miRNAs. Further, to identify whether this stem core region is necessary for miR159a.1 and miR159a.2 regulations, we created a construct by removing the stem region. Notably, in all the above constructions, the miRNA secondary structures were kept almost similar to wild type except changes the positions of precursor miRNA. Finally, we completely destroyed the secondary structure of pri-miR159a by repeating the terminal loop sequence, miR159a.3 and also deleted some base pairs from miR159a.1 to validate role of the nested secondary structures in miRNA processing. In addition, except *mir159a.123-mix*, others construct had longer sequence length than the wild type pri-miR159a (184 nts; Figure 1B). By following this strategy, we performed the complementation test of these nested structures to recognize the value of each duplex miRNA.

As deregulated MYB33/65 activity by miR159 is tightly correlated with the extent of upward leaf curl (Allen et al., 2007), this trait was used to visually assess the strength of the complementation of the *mir159ab* phenotype by each construct and was categorized using three levels: none, mild, and severe (Figure 1C). Furthermore, rosette diameter, leaf area, lamina length, and lamina width in transgenic plants were examined. By using this strategy, we analysed nested miR159a structure regulation in *A. thaliana*.

### Complementation Analysis in the Extended Proximal Fold Back for Nested pri-miR159a Structure Regulatory Footprints

Since all *mir159ab* defects are caused by the deregulation of the redundant GAMYB-like gene pair *MYB33*/*MYB65*, the *mir159ab* double mutant is an excellent method for investigating miRNA-mediated gene silencing (Allen et al., 2007), and these mutant phenotypes, including leaf curl and dwarfed stature, are easily scored. Therefore, by complementing *mir159ab* with different miR159a variants with modified miRNA duplex positions, their abundance, and deficiency, the impact of asymmetrical miRNA secondary structures on the silencing outcome of miR159 or their variants can be evaluated.

**Figure 2:**
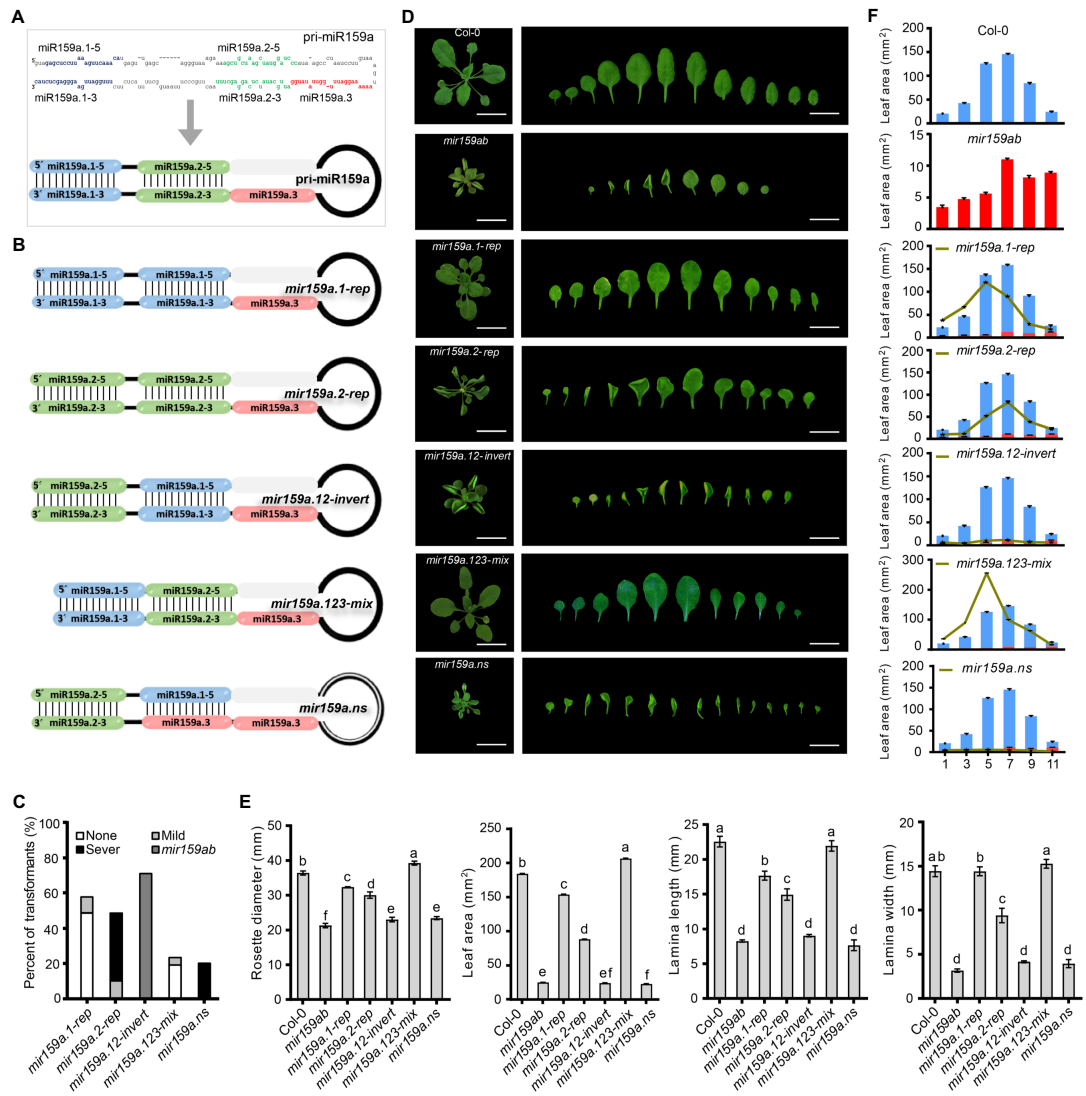
Complementation assay in the extended proximal fold back of nested pri-miR159a structure. **(A)** Schematic showing the sequence and structure design for pri-miR159a. **(B)** Nested structural variant design in the extended proximal fold back of pri-miR159a-driven transgenic Arabidopsis. **(C)** Percentage of primary transformants showing no, mild, and severe leaf curl phenotypes. **(D)** Phenotypes of 22-day-old transgenic plants. Scale bar: 1 cm. **(E)** The size of a rosette, lamina length, lamina width, and leaf area of plants were evaluated. For measurement, the fourth true leaf of 28-day-old plants was collected. Data are presented as the mean ± SD (Standard deviation; *n* > 10) and statistically distinct genotypes were determined by one-way ANOVA with a *post-hoc* least significant difference (LSD) multiple comparisons within each group (*p* < 0.05, marked with different characters). **(F)** Leaf area of (1st, 3rd, 5th, 7th, 9th, and 11th) plants was evaluated. For measurement, 28-day-old plants were collected. Data are presented as the mean ± SD (Standard deviation; *n* > 5).

**Figure 3:**
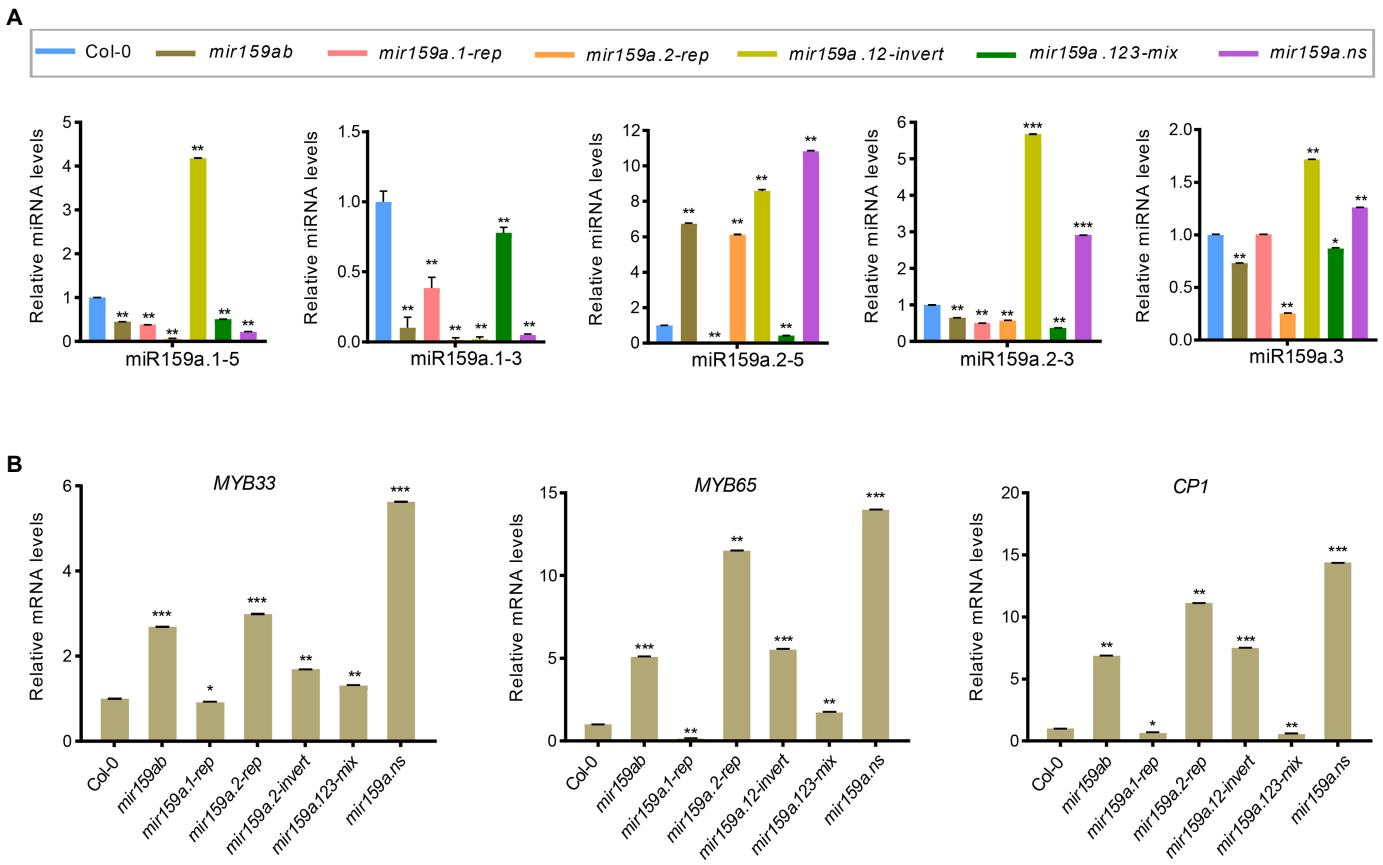
Expression analysis of miR159a sibling’s miRNA and MYB genes in the extended proximal nested transgenic plants. **(A)** The miRNA levels of miR159a siblings in the rosettes of *mir159ab* plants complemented by the nested miR159a variants in comparison with wild type and *mir159ab*. All miR159a siblings and mature miR159a levels were normalized to *U-6* and the relative expression in the wild type was set as 1. Measurements are the average of three technical replicates. Error bars represent the SEM (Standard error mean). Asterisks indicate statistically significant differences at *p* < 0.05 (^*^) and *p* < 0.01 (^**^) by Student’s *t*-test. **(B)** The mRNA levels of *MYB33*, *MYB65*, and *CP1* were examined in Col-0, mir159ab and nested miR159a variants and were normalized to *CYCLOPHILIN*. The relative expression in the wild type was set as 1. Measurements are the average of three technical replicates. Error bars represent the SEM (Standard error mean). Asterisks indicate statistically significant differences at *p* < 0.05 (^*^) and *p* < 0.01 (^**^) by Student’s *t*-test. ^***^ mean *p* < 0.001.

For the study of nested structure regulation in the extended fold back regions, we artificially designed a total of 5 constructs (Figures 2A,B) by repeating (*mir159a.1-rep*, *mir159a.2-rep*), inverting (*mir159a.12-invert*), and deleting (*mir159a.123-mix, mir159a.ns*) the miR159a duplex precursor. Among them, four constructs were symmetrical, and one had an asymmetrical structure in which the miR159a secondary structure was disrupted by repeating miR159a.3 and a terminal loop. All the constructs were named according to their structural changes as follows: (I) *mir159a.1-rep*, in which the miR159a.2 duplex region is replaced with an miR159a.1 duplex; (II) *mir159a.2-rep*, in which the mir159a.1 duplex region is replaced with an mir159a.2 duplex; (III) *mir159a.12-Invert*, in which the miR159a.1 and miR159a.2 duplex regions are mutually exchanged; (IV) *mir159a.123 mix*, in which the stem region between miR159a.1 and miR159a.2 was deleted and the remaining fold backs were mixed; (V) *mir159a-nested structure*, in which the miR159a secondary structure was disrupted completely as compared to miR159a (Figure 2B). The five miR159a variant constructs were individually transformed into *mir159ab*, and the phenotypes of multiple primary transformants were scored (Figure 2C). Leaf area, leaf length, and leaf width were measured at individual leaf positions of plants harvested 22 days after stratification (DAS), which is just before bolting occurs in both systems. Growth measurements were performed at this time point to decrease the potential influence of flowering time on leaf number and/or size (Figure 2D). From these data, we found that the *mir159a.1-rep* and *mir159a.123* mixed transgenic lines showed a rescued *mir159ab* phenotype. The lamina width was restored to the same level as that of the wild type, and rosette diameter, leaf area, and lamina length were also significantly increased compared to that of *mir159ab* (Figure 2E). Furthermore, the leaf area of 1st, 3rd, 5th, 7th 9th, and 11th leaves in transgenic plants was like that of wild-type and *mir159ab* plants, which also showed significant restoration in *mir159a.1-rep* and *mir159a.123-mix* transgenic plants but not others (Figure 2F). We further detected the expression level of *MIR159* and its response factors *MYB33* and *MYB65* (Figure 3). All 57 *mir159a.1-rep* T1 plants were fully complemented with thick and short wide leaves compared to those of the wild type (Figures 2C,D). Interestingly, the expression of miR159a.1–3 and miR159a.1–5 was not induced in the *miR159a.1-rep* transgenic plants, but the levels of *MYB33* and *MYB65* were still suppressed, which hinted that there was another pathway that was independent of the expression of miR159a.1 and related to the structure of the miR159 precursor, to regulate *MYB33* and *MYB65.* In contrast, all 48 *mir159a.2-rep* primary transformants showed the severe leaf curl phenotype with *mir159ab*, indicating that the introduction of miR159a.2 in place of miR159a.1 and keeping the position of miR159a.2 in the secondary structure made the *mir159ab* phenotype more serious. Mature RNA expression analysis showed that the miR159a.2–5 expression was induced six-fold possibly because of the structure with the double miR159a.2 duplex being transformed into the plant. However, the expression level of miR159a.1 in the *mir159a.2-rep* was not increased, and *MYB33* and *MYB65* were still expressed at a high level so that the phenotype was not rescued. Similarly, *mir159a.12-invert* could not rescue the phenotype, indicating that the miRNA structural position has a potential role in silencing efficacy. To further test whether the stem segment of the long miR159a precursor between the miR159a.1 and miR159a.2 duplex was necessary for its processing, we deleted the stem region of the miR159a precursor (Figures 2C,D). Surprisingly, all 23 *mir159a.123mix* primary transformants appeared similar to the wild type, but their leaves were wider than those of the wild type, and <16% of transgenic plants showed rounded leaves and the rescued phenotype, suggesting that individual absence of core stem does not prevent silencing altogether (Figures 2C,D). Furthermore, we irregularly disrupted the pri-miR159a secondary structure by repetition of miR159a.3 and the terminal loop and found that all 20 *mir159a.ns* primary transformants did not rescue the phenotype of the *mir159ab* mutant (Figures 2C,D), which showed a severe phenotype with male sterility characteristics. Taken together, these results indicate that the structure of the miR159 precursor plays an important role in miRNA function, including influencing the *MIR159* expression level and downstream response factor regulation. We also hypothesize that the miR159a.1 part and where it is in the structure may play more important roles according to the phenotype rescued only in *mir159a.1-rep* and *mir159a.123-mix* transgenic lines but not others. qRT-PCR was then performed on rosettes to measure the expression levels of sibling mature miRNAs in nested miR159a structure transgenic plants. We obtained the differential expression profiles of mature sibling miRNAs (Figure 3A). For instance, except for *mir159a.12invert*, mature complementary miR159a.1–5 and miR159a.1–3 expression was lower in both rescued and nonrescued transgenic plants than in wild-type plants. In contrast, the expression level of miR159a.2–5 was 2- to 10-fold higher in all non-rescued plants than in the wild type. miR159a.2–3 and miR159a.3 showed a similar expression pattern with 1-to-2-fold higher expression in *mir159a.12-invert* and *mir159a.ns* compared to the wild type (Figure 3A). Furthermore, as predicted, the *MYB33* transcript level was elevated 1- to 6-fold in mild- to severe-phenotype mutants, while low transcript levels were present in plants not expressing the phenotype. In addition, *MYB65* and *CP1* were dramatically suppressed with a similar pattern in the none phenotype group (Figure 3B).

### Minimum Sequence Length Required for Accurate Processing of Nested pri-miR159a Structures

**Figure 4:**
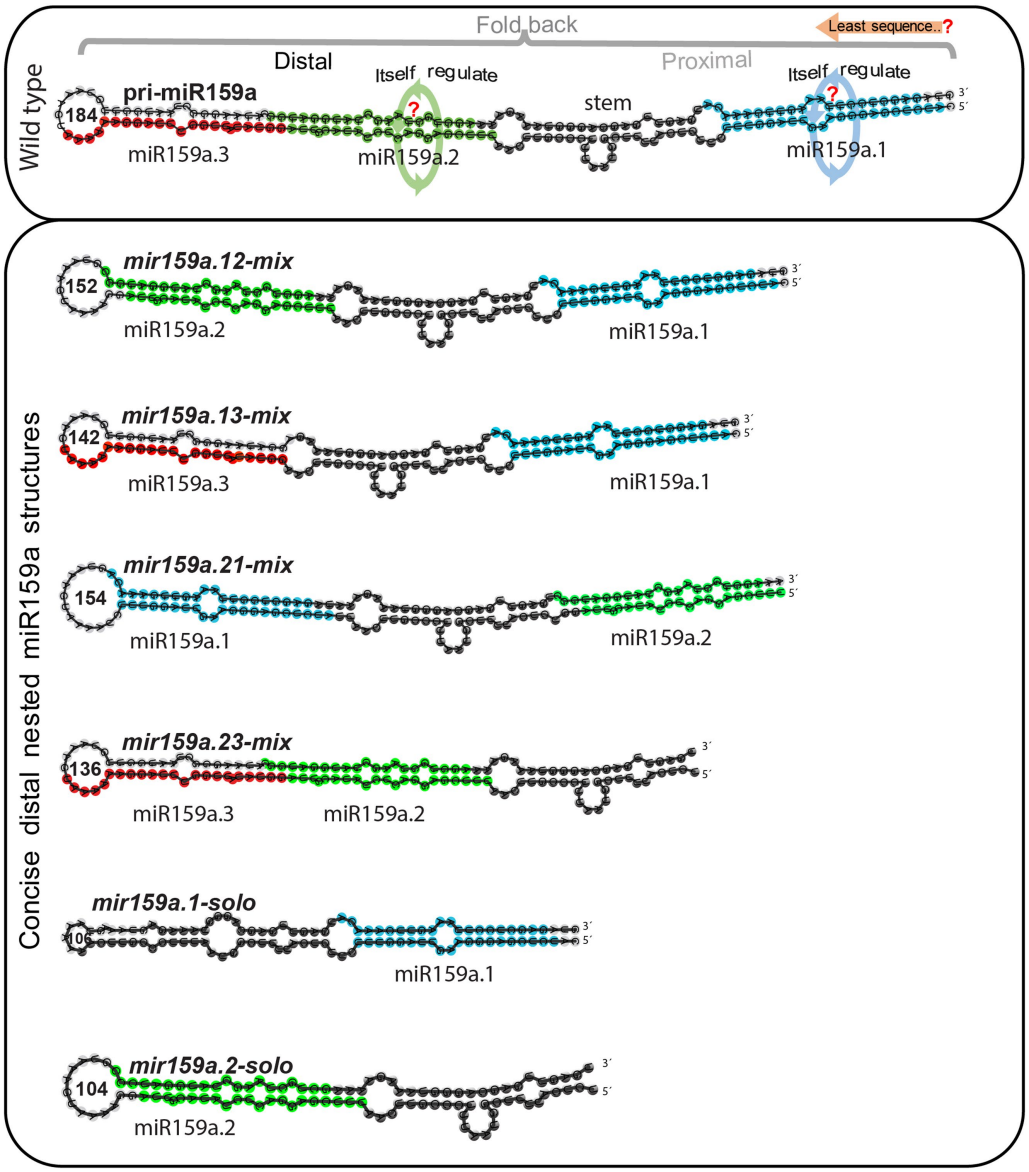
Least sequence required for accurate processing of nested pri-miR159a structures. Schematic secondary structural view for pri-miR159a (upper box) set with comparison to different artificially designed variants containing least sequence (lower box) for complementation test in the concise distal region. Blue, green, red, and black region on miRNA secondary structure indicates, miR159a.1, miR159a.2, miR159a.3, and core stem region, respectively. The orange arrow indicates the minimum sequence requirement for nested miR159a structure regulation. While the green and blue circle with arrow indicates itself regulation of corresponding miRNAs (upper box). Each nested variant is named according to the combination of miRNA duplex region.

The length of mature miRNAs generated from different miRNA genes may differ (Griffiths-Jones et al., 2007). The fast increasing interest in miRNA length diversity expands the miRNA universe and improves the regulatory potential of miRNAs (Chiang et al., 2010). Besides this, the secondary structure of the pri-miRNA has significant implications for miRNA processing (Starega-Roslan et al., 2011). For example, pri-miR172a required an ~15-nucleotide fragment in the lower stem for processing (Mateos et al., 2010; Werner et al., 2010), whereas the upper stem region had only a modest influence. In contrast, the conserved upper stem of pri-miR319, which includes a terminal loop, is important for sequential cleavage for miR319 synthesis, but the lower stem section of pri-miR319 is superfluous (Bologna et al., 2009). Thus, the structural determinants that affect miRNA regulation differ based on the miRNA and miR159a has not yet been formally addressed. Thus, we further investigate the regulation of the nested structure with the minimum sequence length of the pri-miR159a fold back (if the miR159a.1 part is the key sequence) for accurate processing of miR159a, we further artificially designed six symmetrical constructs (Figure 4) by shortening the length of nucleotides ranging from 154 to 104 by mixing, replacing, inverting, and deleting the miR159a duplex region and designated them as (I) *mir159a.12-mix*, in which the miR159a.1 and miR159a.2-duplex regions were combined and miR159a.3 was deleted; (II) *mir159a.13-mix*, in which the miR159a.1 and miR159a.3-duplex regions were combined and the miR159a.2 duplex was deleted; (III) *mir159a.21-mix*, in which the miR159a.1 and miR159a.2-duplex regions were exchanged and miR159a.3 was deleted; (IV) *mir159a.23-mix*, in which the miR159a.1 duplex region was deleted only; (V) *mir159a.1-solo*, in which only the miR159a.1-duplex with a stem part was combined with a terminal loop; (VI) *mir159a.2-solo*, in which the only miR159a.2-duplex region was directly combined with the terminal loop region instead of the stem.

### Complementation Analysis in the Concise Distal Fold Back for Accurate Processing of Nested pri-miR159a Structures

**Figure 5:**
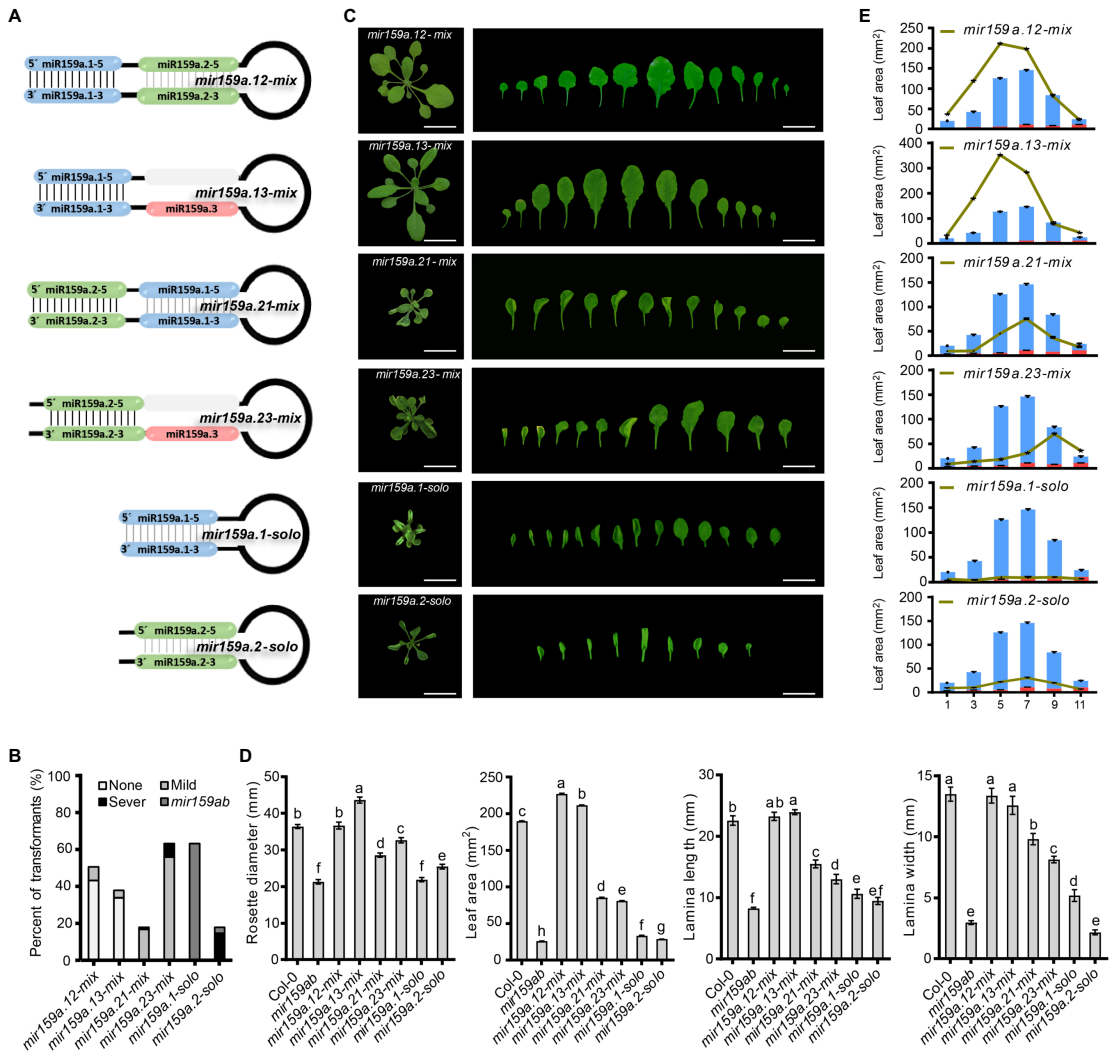
Complementation assay in the concise distal fold back of nested pri-miR159a structure. **(A)** Schematic showing nested structure complementation in the concise distal fold back of pri-miR159a-driven transgenic *Arabidopsis*. **(B)** Percentage of primary transformants showing no, mild, and severe leaf curl phenotypes. **(C)** Phenotypes of 22-day-old transgenic plants. Scale bar: 1 cm. **(D)** The size of a rosette, lamina length, lamina width, and leaf area of plants were evaluated. For measurement, the fourth true leaf of 28-day-old plants was collected. Data are presented as the mean ± SD (Standard deviation; *n* > 10) and statistically distinct genotypes were determined by one-way ANOVA with a *post-hoc* least significant difference (LSD) multiple comparisons within each group (*p* < 0.05, marked with different characters). **(E)** Leaf area of (1st, 3rd, 5th, 7th, 9th, and 11th) plants was evaluated. For measurement, 28-day-old plants were collected. Data are presented as the mean ± SD (Standard deviation; *n* > 5).

Similarly, in the second level of the nested structure, we shortened the sequence length by deleting the sibling mature miRNA duplex region, and we named them the concise distal pri-miR159a groups (e.g., *mir159a.13*-*mix*, *mir159a.23*-*mix*; Figure 4). These six miR159a constructs were individually transformed into *mir159ab*, and the phenotypes of multiple primary transformants were scored. As all 48 *mir159a.12-mix* T1 plants were fully complemented with a large petiole, their rosette leaf margins were slightly undulated compared to those of the wild type (Figures 5A−C). Similarly, all 36 *mir159a.13-mix* primary transformants appeared like the wild type, but their leaves were longer than those of the wild type with little spiny (with sharp stiff points) rosette leaves (Figures 5A−C), suggesting that individual absence of the core stem and miR159a.2 does not prevent silencing alone. In contrast, all 16 and 42 primary transformants appeared mild from *mir159ab* (Figures 5A−C), indicating that the introduction of miR159a.2 in place of miR159a.1 and keeping the position of miR159a.2 in the secondary structure can prevent complementation of the *mir159ab* phenotype and act as a measure of silencing. Similarly, *mir159a.1-solo* and *mir159a.2-solo* analysis indicated that all 60 and 17 primary transformants did not rescue the phenotype of the *mir159ab* mutant (Figures 5A−C), indicating that the miRNA structural position has a potential role in silencing efficacy. The rosette diameter, lamina length, lamina width, and leaf area were also significantly restored to the wild-type values in *mir159a.12-mix* and *mir159a.13-mix* transgenic plants but not others (Figures 5D,E).

**Figure 6:**
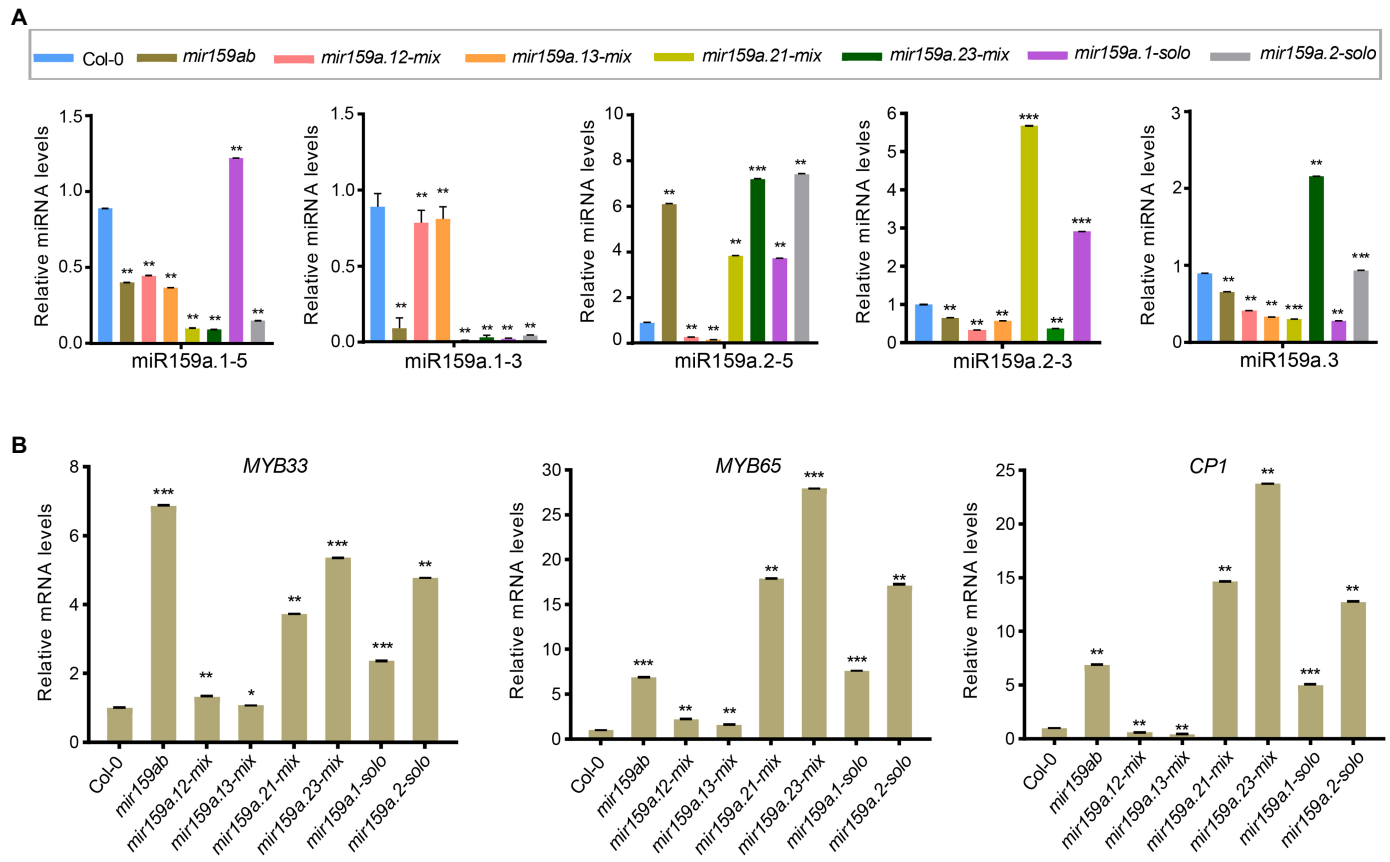
Expression analysis of miR159a sibling’s miRNA and MYB genes in the concise distal nested transgenic plants. **(A)** The miRNA levels of miR159a siblings in the rosettes of *mir159ab* plants complemented by the nested miR159a variants in comparison with wild type and *mir159ab.* All miR159a siblings and mature miR159a levels were normalized to *U-6* and the relative expression in the wild type was set as 1. Measurements are the average of three technical replicates. Error bars represent the SEM (Standard error mean). Asterisks indicate statistically significant differences at *p* < 0.05 (^*^) and *p* < 0.01 (^**^) by Student’s *t*-test. **(B)** The mRNA levels of *MYB33*, *MYB65*, and *CP1* were examined in Col-0, *mir159ab* and nested miR159a variants and were normalized to *CYCLOPHILIN.* The relative expression in the wild type was set as 1. Measurements are the average of three technical replicates. Error bars represent the SEM (Standard error mean). Asterisks indicate statistically significant differences at *p* < 0.05 (^*^) and *p* < 0.01 (^**^) by Student’s *t*-test. ^***^ mean *p* < 0.001.

To investigate whether miRNA abundances are affected by structural determinants that are important for miR159a processing in the nested concise fold, miRNA assays were performed to estimate sibling mature miR159 levels. Similar to the extended fold back, we also obtained the differential expression profiles of mature sibling miRNAs (Figures 6A,B). The same contradictory results showed that although the phenotype was rescued in *mir159a.12-mix* and *mir159a.13-mix* transgenic plants, the expression level of miR159a.1 was not increased, and the transcript levels of *MYB33*/*MYB65* and *CP1* were reduced compared to that of *mir159ab*. In contrast, miR159a.1–5 expression was higher in only *mir159a.1-solo*, but the phenotype was maintained, and the transcript levels of *MYB33*/*MYB65* and *CP1* were still high. These results suggested that there existed another way that self-supported the expression of miR159a.1 and was related to the structure of the miR159 precursor to regulate the target mRNA (Figures 6A,B).

## Discussion

In plants, many miRNA-target relationships are ancient, and they appear to play fundamental roles in plant growth and development. Despite extensive analyses, there has been little investigation into the properties of structural determinants that govern their efficacy. Here, taking advantage of the miR159-MYB33/MYB65 module as a model system, we examined the new nested miR159 structure properties that control the efficacy of silencing by a highly conserved plant miRNA.

A previous study reported that RNA secondary structures are the major determinant of the silencing efficacy of miR159 in Arabidopsis and contribute to the narrow functional specificity of miR159 (Allen et al., 2007; Li et al., 2014; Zheng et al., 2017). To further narrow down this miR159 functional specificity, we functionally analysed the effect of nested miR159a secondary structures to identify structural determinants important for miR159 processing. Here, we introduced a series of nested structure duplex precursor mutations that either disrupted base pairing or closed unpaired bases in the foldback to identify structural features important for miRNA regulation.

**Figure 7:**
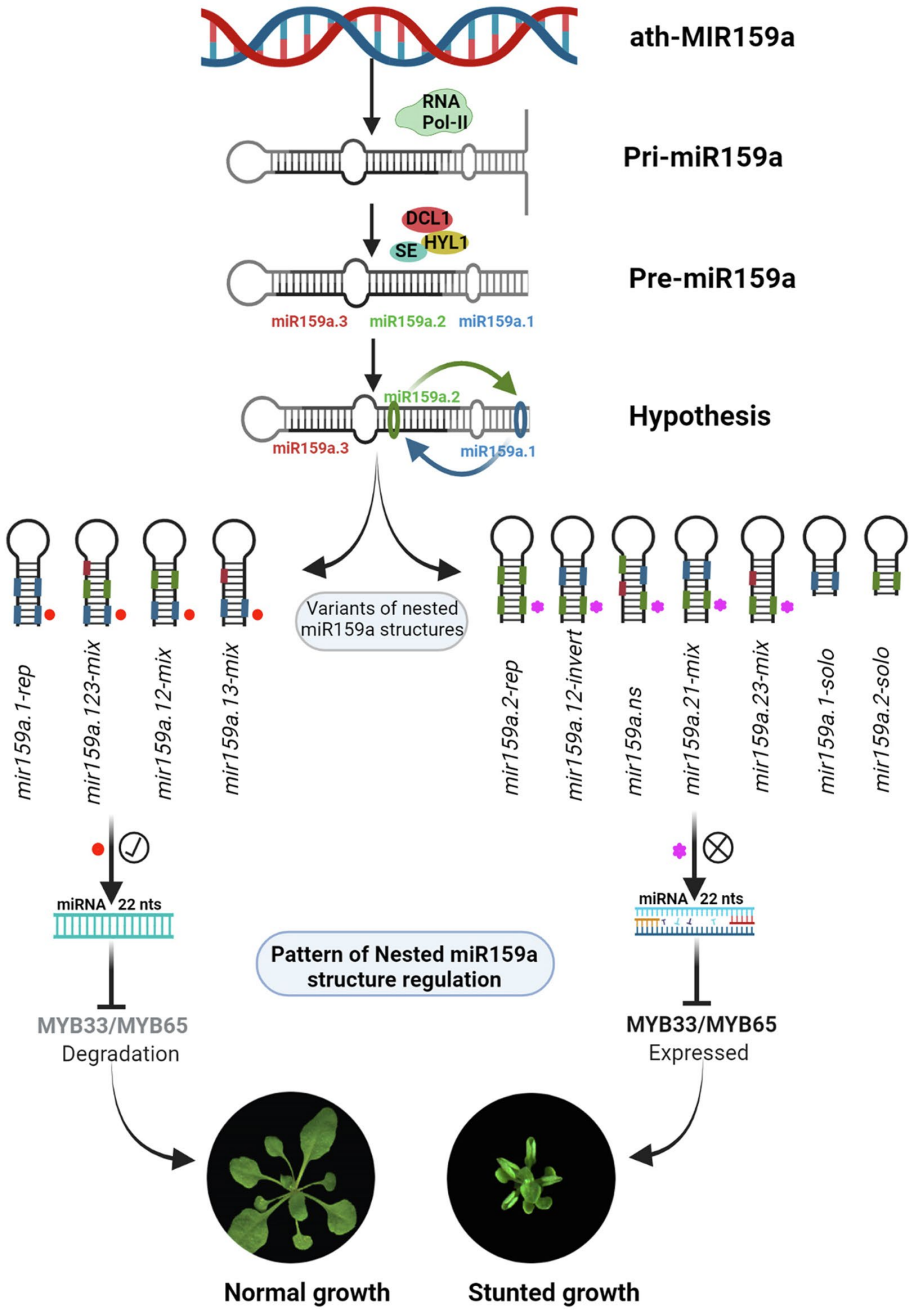
The schematic representation of nested miR159a structure regulation in *Arabidopsis thaliana.*

In our study, we found that *mir159a.1-rep* and *mir159a.123-mix* from extended proximal parts (Figure 2) and *mir159a.12-mix* and *mir159a.13-mix* from concise distal parts (Figure 5) have a miR159a.1 duplex structure at the tail end of pri-miR159a (Figure 7) and rescued the phenotype with little differences in morphology. Additionally, the expression level of miR159a.1–3 was increased compared to that of *mir159ab*, whilethe transcript levels of *MYB33/MYB65* were almost similar/little higher compared to wild type Col-0. These results suggested that the miR159a.1 duplex present at the tail end region may result in an equal function in the regulation of plant development. However, we transformed the miR159a.1 duplex only from the concise distal part (*mir159a.1-solo*) and observed that the primary transformants did not show a rescued phenotype and that the majority showed severe morphology, that further suggests that a specific sequence length is required to work properly It has been reported that plants possess a wider range in the length of pri-miRNA than do animals, which indicates that the long stem region is important for miRNA cleavage (Bologna et al., 2013a), thus supported our results. In plants, the miRNA/miRNA* duplex is generated from two rounds of cleavage of the DCL1-HYL1-SE complex, and the first cleavage reaction at the lower-stem region is more important for the generation of the special duplex. In our study, the appropriate miR159a.1–3 was only expressed when the miR159a.1 duplex at the tail end of the pri-miR159a included the *mir159a.1-rep*, *mir159a.123-mix*, *mir159a.12-mix* or *mir159a.13-mix*, but the *mir159a.12-invert* constructs failed to rescue the phenotype of the mutants, which is consistent with the importance of the flanking sequence of the lower stem for the first cut of the DCL1-HYL1-SE complex (Bologna et al., 2013a). Furthermore, the results suggested that the upper stem is also necessary for the second cleavage reaction; nevertheless, the reaction was only dependent on the stem length rather than the specific sequence.

In contrast, the *mir159a.2-rep* from the extended proximal part (Figure 2), *mir159a.21-mix*, and *mir159a.23-mix* from the concise distal part (Figure 5) showed a miR159a.2 duplex structure at the tail end of the pri-miR159a fold back, and all the primary transformants did not rescue the phenotype and showed the mild phenotype with little differences in morphology (Figure 7). These results suggest that when a duplex nested structure is present on the tail end, part of the pri-miR159a may have a possible dominant function, but after a certain length of the sequence, it may not work properly. Finally, to confirm the effect of the miR159a secondary structure role in processing determinants, a *mir159a.ns* construct was designed with a disrupted miRNA secondary structure, and we observed that all the primary transformants did not rescue the phenotype and showed a severe phenotype and male sterility characteristics, suggests that an asymmetric duplex differed in unpaired nucleotides with protruding base moieties might alters the RISC programming (Figure 5). It has been reported that the AGO1-RISC activates the machinery involved in siRNA synthesis, and an asymmetric miRNA duplex changes the conformation of AGO1 (Manavella et al., 2012), which further support our result of nested duplex structure regulation. Furthermore, we found that the expression level of miR159a.2–3/2–5 was increased and that miR159a.1–3 was absent in transgenic plants, which suggested that miR159a.1 may play a negative role in regulating the expression of miR159a.2. Although the higher expressed miR159a.2 could not rescue the phenotype of the mutants, most of the transgenic lines showed a mild phenotype and the transcript levels of MYB33/MYB65 were significantly higher compared to wild type Col-0 indicating that miR159a.2 may function in some developmental process. It will be a challenge to determine the role of miR159a.2 in further studies. It is well known that pre-miR159 is processed through the non-conical pathway, and their biogenesis starts from loop to base, while in contrast, pri-miR172a processes from the base to the loop of the fold-back structure (Bologna et al., 2013b). Therefore, it is likely that two processing pathways for evolutionarily conserved miRNAs exist in plants. Similarly, these duplex nested structures may have more possible processing pathways, but further investigation is needed to confirm these results.

Overall, our results revealed the miRNA abundances in the nested MIRNA transformants with severe phenotypic defects were higher than those of miR159a in wild-type plants, and wild-type plants had approximately four-fold higher miR159 levels than the miR159a mutant in which *MYB33*/*65* was fully suppressed. This suggests that the silencing effectiveness of miR159a could be an order of magnitude greater than that of all of the other nested miRNAs studied. Furthermore, the continuously higher expression of the miR159a.2 duplex suggests that miR159a.2 is functional in *Arabidopsis* and that its accumulation is completely different from that of miR159a.1, implying that they may have regulatory roles that are independent of one another. This arrangement is conserved across plant populations, with some accumulating miR159.2 in visible quantities, inferring those similar processes could be at work in other plants.

## Conclusion

In brief, these findings provide a clue to help decode the information embedded in the sequence of the pri-miRNA duplex region in response to nested structure regulation of miRNAs.

## Data Availability Statement

The original contributions presented in the study are included in the article/Supplementary Material; further inquiries can be directed to the corresponding author.

## Author Contributions

ZT designed the experiments and managed the project. MI, TL, ZW, MW, SL, XG, AW, and SFL performed gene cloning and functional analysis. MI, MZ, and ZT wrote the manuscript. All authors contributed to the article and approved the submitted version.

## Funding

This work was supported by grants from the National Key Research and Development Program of China (2021YFF1000101) and the International Partnership Program of Chinese Academy of Sciences (153E11KYSB20190045).

## Conflict of Interest

The authors declare that the research was conducted in the absence of any commercial or financial relationships that could be construed as a potential conflict of interest.

## Publisher’s Note

All claims expressed in this article are solely those of the authors and do not necessarily represent those of their affiliated organizations, or those of the publisher, the editors and the reviewers. Any product that may be evaluated in this article, or claim that may be made by its manufacturer, is not guaranteed or endorsed by the publisher.

## Footnotes

^1^ http://rsb.info.nih.gov/ij/
